# 1873. Comparative performance of RTqPCR vs RTddPCR for the detection of SARS-CoV-2 in wastewater (WW) collected from a range of sites and scales across the sewer network of Calgary, Alberta

**DOI:** 10.1093/ofid/ofac492.1500

**Published:** 2022-12-15

**Authors:** Barbara Jean M. Waddell, Lisa Oberding, Nicole Acosta, Maria Bautista Chavarriaga, Janine McCalder, Noah Toppings, Kristine Du, Puja Pradhan, Navid Sedaghat, Alexander Beaudet, Lawrence Man, Jason Cabaj, Srijak Bhatnagar, Norma J Ruecker, Gopal Achari, M Cathryn Ryan, Jon Meddings, John M Conly, Kevin Frankowski, Casey R J Hubert, Dylan Pillai, Michael Parkins

**Affiliations:** University of Calgary, Calgary, Alberta, Canada; University of Calgary, Calgary, Alberta, Canada; University of Calgary, Calgary, Alberta, Canada; University of Calgary, Calgary, Alberta, Canada; University of Calgary, Calgary, Alberta, Canada; University of Calgary, Calgary, Alberta, Canada; University of Calgary, Calgary, Alberta, Canada; University of Calgary, Calgary, Alberta, Canada; University of Calgary, Calgary, Alberta, Canada; University of Calgary, Calgary, Alberta, Canada; University of Calgary, Calgary, Alberta, Canada; University of Calgary, Calgary, Alberta, Canada; University of Calgary, Calgary, Alberta, Canada; Calgary City, Calgary, Alberta, Canada; University of Calgary, Calgary, Alberta, Canada; University of Calgary, Calgary, Alberta, Canada; University of Calgary, Calgary, Alberta, Canada; University of Calgary, Calgary, Alberta, Canada; University of Calgary, Calgary, Alberta, Canada; University of Calgary, Calgary, Alberta, Canada; University of Calgary, Calgary, Alberta, Canada; University of Calgary, Calgary, Alberta, Canada

## Abstract

**Background:**

We sought to compare WW SARS-CoV-2 RNA detection across a range of sites and scales using RTqPCR and RTddPCR.

**Figure.**
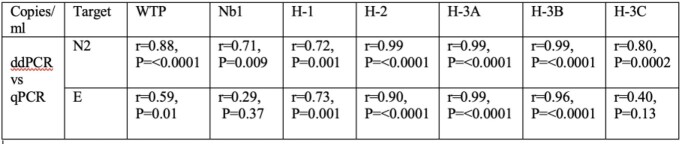

**Methods:**

Composite-24h WW was collected from a WW treatment plant (WTP; n=18), a neighborhood (Nb1; n=12) and three hospitals; H-1, H-2, and H-3 (3-sites; A-C)(n=84). RNA was extracted using the 4S-silica column method. RTqPCR (QuantStudio5, ThermoFisher) and RTddPCR (C1000 Thermal Cycler and QX200 Droplet Reader, BioRad) quantified SARS-CoV-2 RNA nucleocapsid (N2, US CDC) and envelope (E Sarbeco, Corman et al 2020) in triplicate. Fisher’s exact test was used to compare assay sensitivity. Correlations between modalities and RNA - clinically-confirmed COVID-19 cases (defined by postal code of primary residence using 5-day rolling average) was assessed using Persons correlation.

**Results:**

114 samples were tested (02/23/2021-04/22/2021). SARS-CoV-2-N2 was identified in 90/114 (79%) by RTqPCR and 89/114 (78%) by ddPCR (p=1). SARS-CoV-2 E was found in 72/114 (63%) by RTqPCR and 90/114 (79%) by ddPCR, p=0.01. Correlations between modalities were strongest for N2 relative to E across all sites (see Table).

N2 correlated with clinically diagnosed cases for both modalities greater at the level of the WTP (RTqPCR; r=0.8972, p< 0.0001and ddPCR; 0.933, p< 0.0001) relative to neighborhood (RTqPCR; r=0.6, p=0.04 and ddPCR; 0.60, p=0.04). E correlated to a lesser degree with cases at WTP (RTqPCR; r=0.65, p=0.0035 and ddPCR; 0.88, p=< 0.001) and neighborhoods (RTqPCR; r=0.40, p=0.20 and ddPCR; r=0.43, p=0.16).

**Conclusion:**

SARS-CoV-2 detection of N2 was similar between RTqPCR and RTddPCR across a range of sites and scales in the sewershed, and this correlated best with clinical cases whereas E detection was superior with ddPCR.

**Disclosures:**

**All Authors**: No reported disclosures.

